# Plasma Levels of sFas-sFasL and *FASL* Gene Expression Are Associated with Tuberculosis

**DOI:** 10.3390/biom13010080

**Published:** 2022-12-30

**Authors:** Iury de Paula Souza, Ednelza da Silva Graça Amoras, Francisca Dayse Martins de Sousa, Paulo Victor Negrão Raiol de Sousa, Sandra Souza Lima, Izaura Maria Vieira Cayres-Vallinoto, Ricardo Ishak, Antonio Carlos Rosário Vallinoto, Maria Alice Freitas Queiroz

**Affiliations:** 1 Laboratory of Virology, Institute of Biological Sciences, Federal University of Pará (UFPA), Belém 66075-110, Brazil; 2 Graduate Program in Biology of Infectious and Parasitic Agents, Institute of Biological Sciences, Federal University of Pará (UFPA), Belém 66075-110, Brazil

**Keywords:** *Mycobacterium tuberculosis*, tuberculosis, FasL, sFas, sFasL, apoptosis

## Abstract

Apoptosis of macrophages infected by *Mycobacterium tuberculosis* via Fas-FasL is an important immune mechanism against infection. This study investigated the association of tuberculosis (TB) with the presence of the polymorphisms *FAS* -670A/G and *FASL* -124A/G, the levels of sFas and sFasL, and the gene expression of *FASL* and cytokines. Samples of 200 individuals diagnosed with TB and 200 healthy controls were evaluated. Real-time PCR (genotyping and gene expression) and ELISA (dosages of sFas, sFasL, IFN-γ, and IL-10) tests were performed. There was no association of FAS -670A/G and FASL -124A/G polymorphisms with TB. The TB group exhibited high plasma levels of sFas and reduced plasma levels of sFasL (*p* < 0.05). The correlation analysis between these markers revealed a positive correlation between the levels of sFas and sFasL, sFasL and FASL expression, and between sFas and *FASL* expression (*p* < 0.05). In the TB group, there was a positive correlation between FASL expression and IFN-γ levels and higher levels of IL-10 compared to IFN-γ (*p* < 0.05). High levels of sFas and reduced levels of sFasL and *FASL* expression may contribute to the inhibition of apoptosis in infected cells and represent a possible bacterial resistance resource to maintain the infection.

## 1. Introduction

The World Health Organization (WHO) estimates that 1.7 billion people are infected with *M. tuberculosis* worldwide and at risk of developing the disease. In 2019, approximately 10 million people were diagnosed with tuberculosis (TB). In addition, tuberculosis is the leading cause of death from a single infectious agent [1]. The impact of tuberculosis is also reflected in the underreporting of new cases, since less than two-thirds of the annual incidence is duly recorded—a gap that manifests as one of several threats to global health [2].

The most common form of active disease or infection in humans, pulmonary tuberculosis, affects the lungs. However, the bacterium can establish itself in several other organs of the body, characterizing the condition of extrapulmonary tuberculosis. Another form of tuberculosis is latent, which is characterized by the presence of the bacteria in the individual without the development of disease [3].

Infection by *M. tuberculosis* and the appearance of symptoms in susceptible individuals are accompanied by intense immunological signaling by the host, which is initially characterized by the mobilization of mononuclear phagocytes to the infection sites. However, bacterial virulence factors can allow for and prolong the survival of the pathogen inside phagocytic cells, thereby diminishing the bactericidal effects of immune responses [4,5,6].

The presentation of *M. tuberculosis* antigens elicits pro- (Th1) and anti-inflammatory (Th2) responses and stimulates CD8+ T lymphocytes. Once activated, these cells begin to secrete IFN-γ and Th1 profile cytokines, which are essential for infection control, and induce the apoptosis of infected phagocytes [7]. The cytotoxic action of lymphocytes is established through different systems, including programmed cell death, which is activated by the interaction between Fas and its ligand FasL. FasL (or CD95 L), after binding to its Fas receptor (also known as CD95 or APO-I), triggers an apoptosis-signaling cascade in the target cell [8]. Despite the efficiency of this system, *M. tuberculosis* seems to be able to modulate the apoptosis of infected phagocytes in an escape mechanism of an important host defense strategy against *M. tuberculosis* [9,10].

Soluble forms of Fas (sFas) and FasL (sFasL) can be produced in different ways. Soluble sFas is the product of the alternative splicing of the pre-mRNA Fas, which is commonly created by the exclusion of transmembrane sequences encoded in exon 6. The loss of these sequences causes molecule inactivation, resulting in a nonfunctional protein that inhibits apoptosis [11]. sFasL originates from the processing of the FasL transmembrane region, mainly by a metalloproteinase [12]. The role of sFasL in apoptosis is still contradictory. In some cases, sFasL is associated with the inhibition of apoptosis activation, and in others, it is considered to be a proapoptotic molecule [12,13,14,15].

Polymorphisms in the *FAS* and *FASL* genes are associated with functional changes in the encoded proteins; among the polymorphisms studied, rs1800682 (*FAS* -670A/G) and rs5030772 (*FASL* -124A/G) were evaluated in the development of neoplasms [16,17] and autoimmune inflammatory diseases [18,19,20], respectively. However, to date, there are no data on the relationship of these polymorphisms with *M. tuberculosis* infection or on their impact on the production of sFas and sFasL. Therefore, the present study investigated the influence of *FAS* -670A/G and *FASL* -124A/G polymorphisms on *M. tuberculosis* infection, the synthesis of sFas and sFasL molecules, and the gene expression of *FASL*, as well as the role of these molecules in the activation of apoptosis in tuberculosis and in possible resistance to infection.

## 2. Materials and Methods

### 2.1. Sample Collection and Characterization

The study performed was observational and cross-sectional and included 200 patients diagnosed with TB at the João de Barros Barreto University Hospital (Hospital Universitário João de Barros Barreto—HUJBB) (patients subjected to screening comprised the outpatient spontaneous demand and inpatients) and the Municipal Health Unit of Guamá—UMS/Guamá (patients subjected to screening comprised the outpatient spontaneous demand). All patients were being treated for tuberculosis for a period ranging from 1 to 2 months.

The inclusion criteria consisted of individuals confirmed positive for tuberculosis after sputum smear microscopy and/or bronchial lavage and culture specific for *M. tuberculosis*, 18 years of age or older, and without coinfection with HIV-1. Patients with diagnosed autoimmune disease were excluded.

A group of 200 individuals who had contact with patients with tuberculosis but who did not develop the disease (older than 18 years old, negative for TB and HIV-1, and without autoimmune diseases) was used to compare the genotype and allele frequencies with the group with tuberculosis. Individuals in the control group were negative for tuberculosis (TB) disease and self-reported having had contact with TB patients (family members or a close person). All subjects in the control group underwent a tuberculin skin test (TST) and had a negative sputum smear and a normal chest X-ray. The TST showed that 95 individuals (47.5%) had a reactive result, while 105 (52.5%) were not reactive for the test. For the comparison of plasma levels of sFas, sFasL, and *FASL* expression, the control group included only subjects with a positive TST.

Whole blood samples (10 mL) were collected in a vacuum collection system in tubes containing ethylenediaminetetraacetic acid (EDTA) as an anticoagulant and sent to the Laboratory of Virology of the Institute of Biological Sciences of the Federal University of Pará (LabVir—ICB/UFPA), where they were separated into cell and plasma mass fractions and stored at −20 °C until use.

### 2.2. DNA Extraction

DNA was extracted from leukocytes of whole blood using the Puregene™ kit (Gentra Systems Inc., Minneapolis, MN, USA) according to the manufacturer’s protocol, which includes steps such as cell lysis, protein precipitation, precipitation, and DNA hydration.

After extraction, the DNA obtained was quantified by spectrophotometric reading using BioDrop™ equipment (Bio-Rad, Hercules, CA, USA) following the protocol recommended by the manufacturer.

### 2.3. Genotyping of FAS -670A/G (rs1800682) and FASL -124A/G (rs5030772)

The identification of the polymorphism genotypes was performed by real-time PCR using the StepOnePLUS™ Real-Time PCR System (Thermo Fisher, Carlsbad, CA, USA). The reactions consisted of the use of commercially obtained TaqMan™ assays—FAS rs1800682 (C_9578811_10) and FASL rs5030772 (C_32334221_10)—containing primers and probes specific for the amplification of the target sequence (Thermo Fisher, Carlsbad, CA, USA). The reaction contained 1× MasterMix, H_2_O, 20× assay C_11537906_20, and 50 ng of DNA, and was subjected to the following cycling conditions: 10 min at 95 °C and 40 cycles of 15 s at 95 °C and 1 min at 60 °C.

### 2.4. Plasma Quantification of sFas and sFasL

The plasma levels of sFas and sFasL were measured by ELISA immunoenzymatic tests: Human Fas ELISA Kit and Human FAS Ligand ELISA Kit (CD95 L) (Abcam, Cambridge, UK). These tests use specific monoclonal antibodies to detect the proteins and were performed according to the manufacturer’s instructions.

### 2.5. RNA Extraction

Total RNA extraction was performed from peripheral blood leukocytes using the RNA Purification Kit (Norgen Biotek Corporation^®^, Thorold, ON, Canada), and all steps followed the protocol recommended by the manufacturer. After extraction, the degree of purity of the sample was determined by the ratio between the absorbances measured at 260 and 280 nanometers (nm), and those with values between 1.6 and 1.8 and with good integrity were selected. The concentration of extracted RNA was determined using the Qubit^®^ 2.0 Fluorometer (Invitrogen, Carlsbad, CA, USA) and the Qubit^®^ RNA HS Assay kit (Invitrogen, Carlsbad, CA, USA), according to the manufacturer’s instructions. All total RNA samples had concentrations equal to 60 nanograms/microliter (ng/µL) and were stored in a freezer at −70 °C until reverse transcription reactions were performed for the synthesis of complementary DNA (cDNA).

### 2.6. Reverse Transcription

The extracted RNA was converted into cDNA using High-Capacity cDNA Reverse Transcription with an RNAse Inhibitor kit (Applied Biosystems, Foster City, CA, USA). For the cDNA reaction, a mixture was prepared with a final volume of 20.0 μL, containing 2 µL of 10× RT Buffer; 0.8 µL of 25× dNTP Mix (100 nM); 2 µL of 10× RT Random Primers; 1 µL of MultiScribe^TM^ (Thermo Fisher, Carlsbad, CA, USA) Reverse Transcriptase; 1 µL of RNaseOUT^TM^ (Thermo Fisher, Carlsbad, CA, USA); 3.2 µL of ultrapure water, provided in the kit; and 10.0 μL of extracted RNA. Subsequently, the mixture was placed in an Eppendorf Mastercycler thermocycler (Eppendorf, Hamburg, Germany) and subjected to cycling at 25 °C for 10 min, 37 °C for 120 min, and 85 °C for 5 min.

### 2.7. Gene Expression

Initially, standardization of the qPCRs with the cDNAs and probes (endogenous genes and target genes) was performed to calculate the efficiency of the amplification reactions. In the standardization reactions, different concentrations of cDNA (pure and in 4 dilutions of Factor 2, 1:2, 1:4, 1:8, and 1:16) were tested. All reactions were performed in plates and in triplicate, and the same cDNA (at different dilutions) was analyzed simultaneously with the different probes to construct an efficiency curve to validate the 2^−ΔΔCT^ analysis method. All tests had an efficiency of 100% (±10) [21].

The real-time PCR for the relative quantification of gene expression consisted of the amplification of the target genes, together with the endogenous gene (normalizer), using TaqMan^TM^ assays (Applied Biosystems, Foster City, CA, USA) and the StepOnePLUS™ Real-Time PCR System (Applied Biosystems, Foster City, CA, USA), and each reaction was performed in a separate well (singleplex) (ThermoFisher, Carlsbad, CA, USA), according to the manufacturer’s protocol. For the FASL gene expression assay, the commercially available TaqMan Gene Expression Assay (Hs00181226_g1) (ThermoFisher, Carlsbad, CA, USA) was used. Each reaction consisted of 15 μL of 2× TaqMan^®^ Universal PCR Master Mix, 1.5 μL of 20× TaqMan Gene Expression Assays, 3 μL of cDNA, and 10.5 μL of RNase-free water. The endogenous gene (reference) was glyceraldehyde-3-phosphate dehydrogenase (GAPDH) detected using the specific Hs02786624_g1 assay (ThermoFisher, Carlsbad, CA, USA). The following thermocycling conditions were used: 2 min at 50 °C, followed by 10 min at 95 °C, 40 cycles of 15 s at 95 °C, and 1 min at 60 °C.

The relative quantification (RQ) of the gene expression of the target genes was determined using the comparative CT method (ΔΔCT) with the Formula 2^−ΔΔCT^, where ΔΔCT = ΔCTsample − ΔCTreference (Life Technologies, Carlsbad, CA, USA).

### 2.8. Plasma Measurement of IFN-γ and IL-10

Plasma levels of IFN-γ and IL-10 were quantified using an enzyme immunoassay (ELISA), IFN gamma Human ELISA Kit, and IL-10 Human ELISA Kit (Thermo Fisher, Carlsbad, CA, USA), which use specific monoclonal antibodies to detect each of the cytokines. The test was performed according to the manufacturer’s instructions.

### 2.9. Tuberculin Skin Test (TST)

Control subjects were subjected to a tuberculin skin test performed by applying an intradermal injection of 0.1 mL (0.04 mcg) of PPD RT-23 (Mantoux, 2 UT/0.1 mL) in the middle third of the anterior surface of the left forearm, at an angle of 5 to 15 degrees, until the formation of a papule. The reading was performed 48 to 72 h after application using a specific millimeter ruler, measuring the largest transverse diameter of the induration perpendicular to the forearm. The results with indurations greater than or equal to 5 mm were considered positive PPD [22].

### 2.10. Statistical Analysis

The information obtained was entered into a database in Microsoft Office Excel 2013 software (Microsoft, Redmond, WA, USA). The determination of the allelic and genotypic frequencies of the polymorphisms was performed by direct counting, and the differences between the groups were evaluated using the chi-square test (χ2). The calculation of Hardy–Weinberg equilibrium was performed to evaluate the distribution of genotypic frequencies. The normality analysis of the association of plasma levels of sFas, sFas, *FASL* gene expression, and cytokines between the studied groups was analyzed using the Shapiro–Wilk test. Thus, the comparisons were performed using nonparametric tests (Mann–Whitney and Kruskal–Wallis). The nonparametric Spearman correlation was used to evaluate the correlation between plasma levels of sFas and sFasL, levels of FASL gene expression, and levels of the cytokines IFN-γ and IL-10. All tests were performed using the software BioEstat 5.3 and GraphPad Prism 5.0, and those with *p* < 0.05 were considered as significant associations.

## 3. Results

The analysis of the distribution of allelic and genotypic frequencies of the polymorphisms in the patients and controls revealed that both groups were in Hardy–Weinberg equilibrium. Comparisons of the genotypic and allelic frequencies of *FAS* -670A/G and *FASL* -124A/G between the TB and control groups showed no significant differences (Table 1).

**Figure 1:**
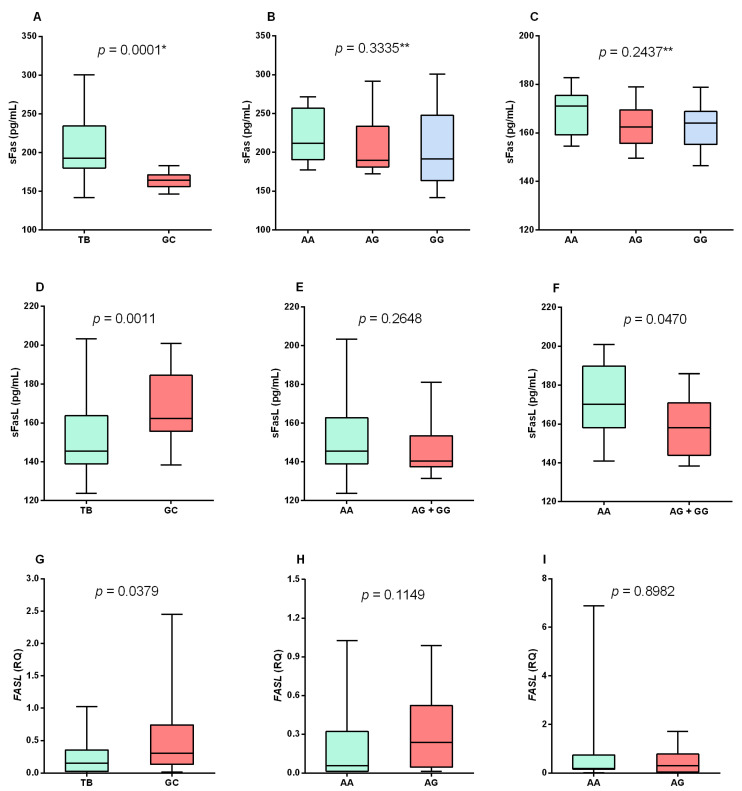
Evaluation of sFas plasma levels between the (**A**) TB and control (GC) groups and the different genotypes for the *FAS* -670A/G polymorphism in the (**B**) TB and (**C**) control groups. Evaluation of sFasL plasma levels between (**D**) TB and control groups and the different genotypes for *FASL* -124A/G polymorphism in (**E**) TB and (**F**) control groups. Evaluation of *FASL* gene expression levels between (**G**) TB and GC groups and the different genotypes for FASL -124A/G polymorphism in (**H**) TB and (**I**) control groups. * Mann–Whitney test; ** Kruskal–Wallis test.

Plasma sFas levels were significantly higher in the TB group than in the control group (*p* = 0.0001; Figure 1A). In contrast, there was no statistical significance in the comparison of the sFas plasma levels between individuals with different *FAS* -670A/G genotypes in the TB group (Figure 1B) and in the control group (Figure 1C).

The plasma levels of sFasL were lower in the TB group than in the control group (*p* = 0.0011; Figure 1D). No significant differences were observed in the plasma levels of sFasL between the patients with different genotypes for the *FASL* -124A/G polymorphism in the TB group (Figure 1E). In the control group, higher levels of sFasL were observed in individuals with the genotype AA than in those with the other genotypes (*p* = 0.0470; Figure 1F).

To confirm the low levels of sFasL, *FASL* gene expression was quantified, which showed that patients with tuberculosis had lower levels of *FASL* mRNA than the control group (*p* = 0.0379; Figure 1G). There was no significant difference in the expression levels between the different genotypes for the *FASL* -124A/G polymorphism in the TB group (Figure 1H) and in the control group (Figure 1I).

**Figure 2:**
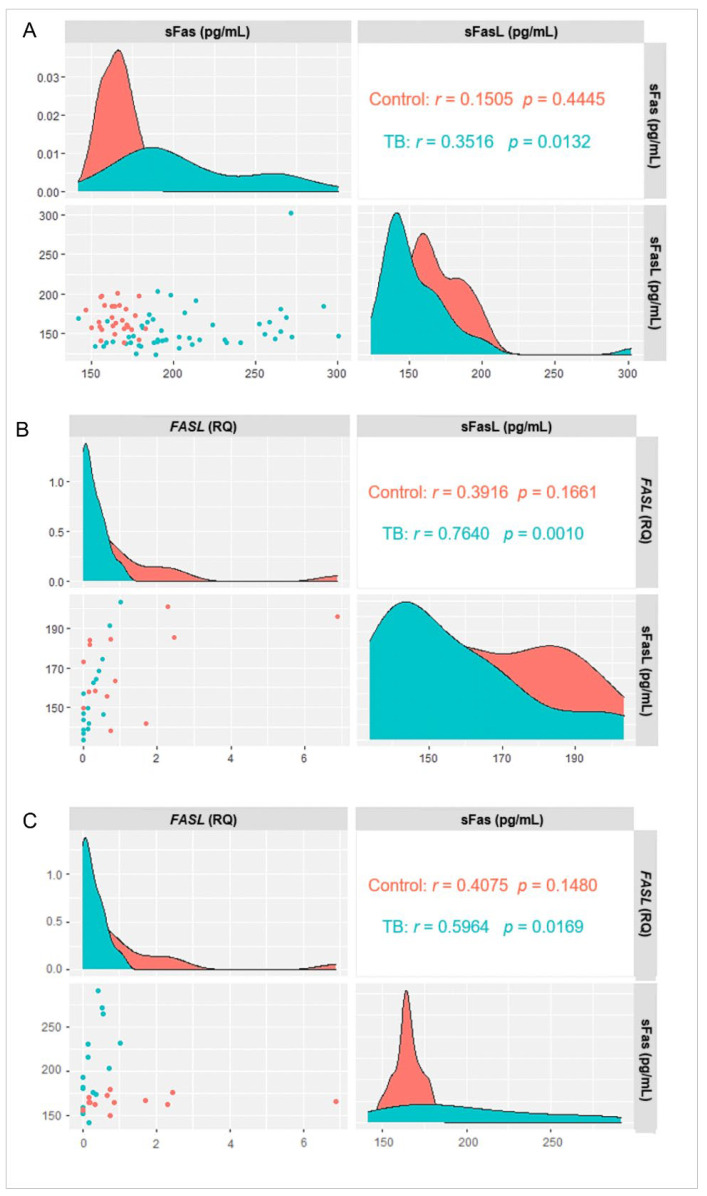
Correlation between (**A**) sFas and sFasL levels, (**B**) sFasL and *FASL* gene expression, and (**C**) sFas and *FASL* gene expression in the TB and control groups. Results determined via Spearman correlation.

The evaluation of the levels of sFas and sFasL showed a positive correlation in the TB group, and no correlation was observed in the control group (*p* = 0.0132; Figure 2A). The correlation was strongly positive between sFasL plasma levels and *FASL* expression in the TB group (*p* = 0.0010; Figure 2B) and between sFas and *FASL* expression levels (*p* = 0.0169; Figure 2C).

**Figure 3:**
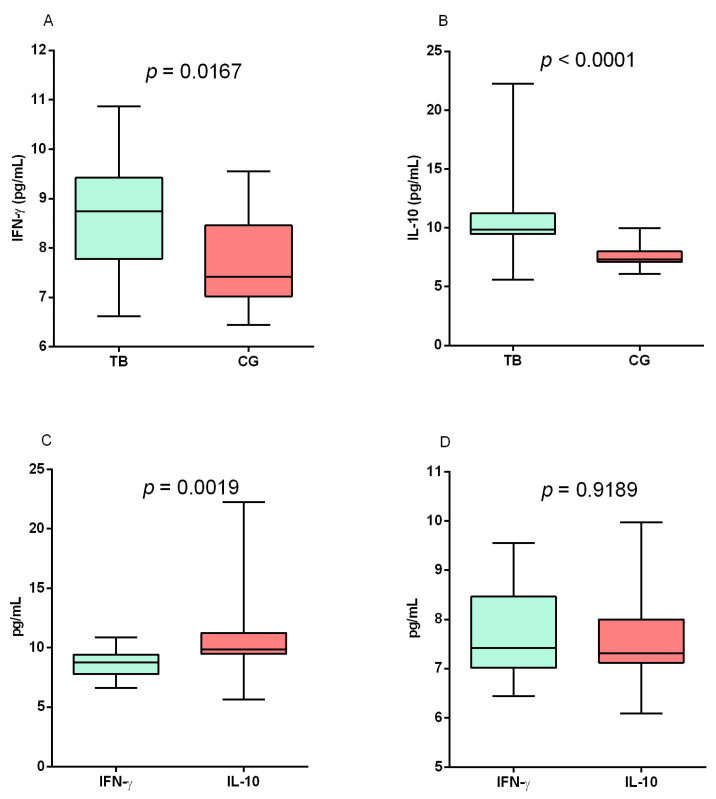
Assessment of (**A**) IFN-γ and (**B**) IL-10 levels between the TB and control (CG) groups. Comparison of IFN-γ and IL-10 levels in the (**C**) TB and (**D**) control groups. Results determined via Mann–Whitney test.

The levels of IFN-γ (Figure 3A; *p* = 0.0167) and IL-10 (*p* < 0.0001; Figure 3B) were significantly higher in the TB group than in the control group. A comparison of the IFN-γ and IL-10 cytokine levels in each of the groups evaluated showed that the IL-10 levels were significantly higher than the IFN-γ levels in the TB group (*p* = 0.0019; Figure 3C), but there were no differences between the levels of both cytokines in the control group (Figure 3D).

**Figure 4:**
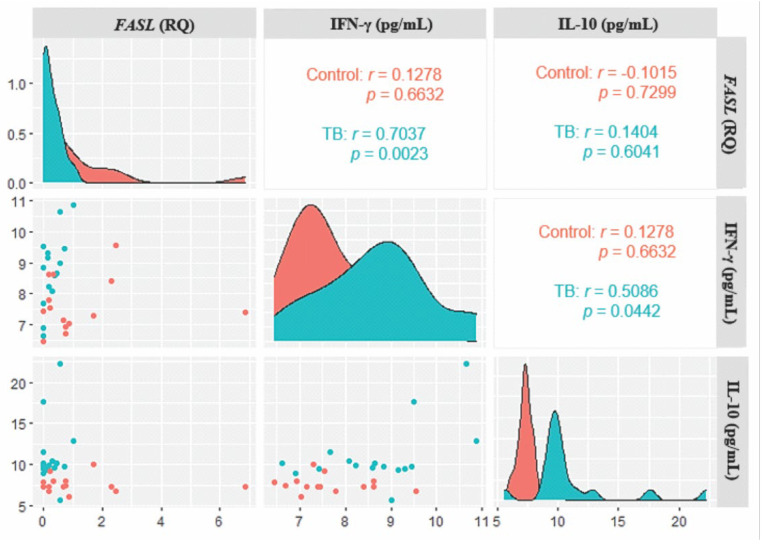
Correlogram of *FASL* expression levels with IFN-γ and IL-10 cytokines in the TB and control groups. Results determined via Spearman Correlation.

In the evaluation of the correlation between the plasma levels of sFas, sFasL, and the levels of gene expression of *FASL* with the cytokines IFN-γ and IL-10, a positive correlation was observed only between the gene expression of FASL and the levels of IFN-γ (*r* = 0.7037; *p* = 0.0023), but there was no correlation with IL-10 levels (Figure 4).

## 4. Discussion

Apoptosis is an important strategy against infection and is affected by signaling cascades that make up a complex network of programmed cell death mechanisms against microbial invasion [23]. However, several microorganisms have developed mechanisms to modulate the activities of host cells; in this context, apoptotic inhibition pathways exert a great influence on the pathogenesis of bacterial diseases [24].

The present study investigated the association of important genetic variations in *FAS* -670A/G and *FASL* -124A/G in patients with tuberculosis and the influence of these polymorphisms on the plasma levels of sFas and sFasL and the gene expression of FASL, since the anti-apoptotic or pro-apoptotic activity of these molecules can determine the progression of *M. tuberculosis* infection and the prognosis of tuberculosis [25,26,27,28,29].

The *FAS* -670A/G and *FASL* -124A/G polymorphisms were associated with autoimmune disease in European and Egyptian subjects [13,19,30] but not with hepatitis B in a population from Iran [31]. The population evaluated in this study is classified as a mixed population, composed of different genetic contributions of white, black, and indigenous people [32]; therefore, it was not possible to evaluate the *FAS* -670A/G and *FASL* -124A/G polymorphisms considering different ethnicities. Thus, our focus was to assess whether the polymorphism would be associated with infection by *M. tuberculosis* and the development of tuberculosis in our population, since other individuals from the same geographic population showed an association with the frequency of the polymorphism *FAS* -670 A/G with HLTV-1 infection and the development of disease symptoms [33], but frequency was unrelated to HBV and HIV infection [34,35]. These results show that the *FAS* -670A/G polymorphism seems to be related to the evolution of infections and the development of specific diseases, not including tuberculosis.

Regarding the plasma quantifications performed, it was observed that the sFas levels were significantly higher in the patients with tuberculosis than in the controls; on the other hand, sFasL levels were significantly lower in the tuberculosis group. sFas is related to the inhibition of the apoptosis process because it can bind to the FasL membrane ligand and inhibit its interaction with membrane Fas, which is responsible for promoting the activation of apoptosis [11,36,37]. In contrast, similar to FasL, sFasL may have proapoptotic activity; therefore, similar to FasL, sFasL also has cytotoxic activity against cells that express Fas, as it promotes the trimerization of Fas molecules and activates apoptosis [13,38,39]. Bajou et al. (2008) showed that Fas was functional when exposed to the sFasL molecule, as it promoted the apoptosis of endothelial cells [15].

As already described, sFas acts as an inhibitor, and sFasL can act as an inducer of apoptosis in different types of disease [36,40,41,42]. Thus, tuberculosis also seems to be associated with this pattern of molecular activities since the present study observed high levels of sFas (anti-apoptotic) and low levels of sFasL (pro-apoptotic). These findings could represent a possible mechanism for the persistence of *M. tuberculosis* and the maintenance of the disease. In a model considering virulent strains of *M. tuberculosis*, they were shown to benefit from the inactivation of apoptosis. Fas expression was shown to be downregulated in macrophages infected by bacillus, making them resistant to this death-signaling pathway [43,44].

The production of sFas is associated with the inhibition of apoptosis in other infectious diseases. In chronic hepatitis C, patients had higher levels of sFas than the control group, which was related to a reduced susceptibility to FasL-induced apoptosis, which may represent an escape mechanism of HCV from immune surveillance [45]. The core protein of hepatitis B virus (HBc) has been implicated in hepatocarcinogenesis through several mechanisms. This protein was associated with the prevention of apoptosis in hepatocytes by reducing the expression of membrane Fas (pro-apoptotic form) and increasing the levels of sFas (anti-apoptotic form), which could contribute to the survival and persistence of hepatocytes infected during a chronic infection [46].

Results similar to those observed in our study regarding the reduced levels of sFasL in patients with TB were demonstrated by Schweizer et al. (2021) in patients with COVID-19 who had low levels of sFasL and were associated with a decrease in membrane Fas signaling and disease severity [47].

Although patients with tuberculosis have high levels of sFas and low levels of sFasL, a significant positive correlation was observed between the levels of sFas and sFasL in this group. To confirm this correlation, the gene expression of FASL was quantified, and its evaluation showed that the expression levels were reduced in the TB group in the same way as the sFasL levels. In addition, the correlation analysis between sFasL and *FASL* was strongly positive, showing that as *FASL* expression increases, sFasL production also increases. The evaluation of the sFas and FASL levels showed a positive correlation, which was similar to the analysis of the sFas and sFasL levels. These results may be related to a possible compensatory attempt of the organism to maintain the active apoptosis pathway. Thus, as the amount of sFas increases, inhibiting apoptosis, it would also be necessary to increase the production of molecules that can reverse the process. However, the increase in *FASL* levels and, consequently, sFasL levels proved insufficient to neutralize the possible antiapoptotic effects of sFas on *M. tuberculosis* infection.

An important observation about the evaluations of the levels of sFAS, sFasL, and *FASL* gene expression is that for these analyses, the control group consisted of individuals with a positive tuberculin skin test (TST) but who never developed tuberculosis. As these individuals had higher levels of sFasL and *FASL*, these preliminary data seem to suggest that the reduction in the levels of these markers in these individuals could indicate a possible progression to active TB. However, this relationship needs to be better investigated through a longitudinal study to follow up on the evolution of latent infection.

The plasma levels of sFas were not associated with any of the genotypes for the polymorphism *FAS* -670A/G. Similarly, the levels of sFasL and *FASL* expression were not related to the *FASL* -124A/G polymorphism. No descriptions were found that evaluated the production of sFas and sFasL in relation to the investigated polymorphisms. According to the data obtained in our study, the *FAS* -670A/G polymorphism does not influence the plasma production of sFas. The *FASL* -124A/G polymorphism was associated with lower levels of sFasL in the control group, but this relationship was not observed in the evaluation of FASL gene expression. As only this group showed a significant difference and we do not have other scientific references with which to evaluate this information, this result may indicate that the polymorphism may influence sFasL levels, but not in the presence of *M. tuberculosis* infection. However, further studies are needed to establish the relationship between *FASL*-124A/G and sFasL levels.

The levels of the cytokines IFN-γ and IL-10 were higher in the TB group, showing that active infection by *M. tuberculosis* induces a strong reactive immune-inflammatory response, which can lead to the activation of different cell populations, characterized by an inflammatory, anti-inflammatory, or regulatory response, which can be specific or nonspecific for the eradication of the infection [48,49,50].

The positive correlation of IFN-γ levels with *FASL* gene expression levels shows the influence of cytokines on apoptosis activation. In T cells, IFN-γ signaling is necessary for the transcription of FasL and its migration to the cell surface, where it can interact with membrane Fas and promote apoptosis, which is considered to be one of the important mechanisms for controlling macrophage infection by mycobacteria [51]. Although higher levels of IFN-γ and lower levels of FASL expression were observed, the correlation between these markers suggests that IFN-γ levels may contribute to influencing the increase in *FASL* expression but not at the same rate that the cytokine is being produced. Furthermore, the maintenance of low levels of *FASL* expression may be a consequence of *M. tuberculosis* interfering with the FASL system in an attempt to avoid the effects of apoptosis [43].

On the other hand, IL-10 levels were higher than IFN-γ levels in the TB group. The cytokine IL-10 is known to suppress Th1 cells and IFN-γ expression. This suppression is achieved by the extrinsic production of IL-10 and through a negative feedback loop that induces an intrinsic expression of IL-10 in cells that also express IFN-γ during the differentiation of the Th1 lineage [52]. Furthermore, high levels of IL-10 were associated with susceptibility to tuberculosis [53]. Thus, the excess production of IL-10 can compromise an efficient Th1 response against *M. tuberculosis* and indirectly contribute to a reduction in the activation of apoptosis in cells infected by the bacillus. However, it is important to emphasize that IL-10 may have differential roles depending on the stage of *M. tuberculosis* infection [49,54,55]. Thus, identifying the stage of infection in which patients are found can provide a better understanding of IL-10’s function.

This study provides relevant information that may contribute to a better understanding of the role of molecules related to the activation/inhibition of apoptosis (sFas, sFasL, and *FASL* gene expression) in tuberculosis. However, the lack of clinical data that characterize the severity of the disease, such as the laterality of lung lesions, the presence of cavities, and the score of radiographic abnormalities, did not allow us to verify whether the levels of apoptosis markers were associated with the severity of tuberculosis; thus, it constitutes a limiting factor of the study.

## 5. Conclusions

In summary, the present study showed that patients with tuberculosis had high levels of sFas and low levels of sFasL and *FASL* gene expression, with a positive correlation between the levels of these markers. The levels of sFas and sFasL and *FASL* expression were not influenced by the *FAS* -670A/G and *FASL* -124A/G polymorphisms, respectively. The tuberculosis patients showed a positive correlation between IFN-γ levels and *FASL* expression levels, but these patients had higher IL-10 levels than IFN-γ levels. The results demonstrate that the immune response developed against *M. tuberculosis* in patients who develop tuberculosis is related to increased levels of sFas (antiapoptotic), reduced levels of sFasL and *FASL* expression (both proapoptotic), and elevated levels of the cytokine IL-10, which may contribute to the persistence of the infection.

## Figures and Tables

**Table 1:** Allelic and genotypic frequencies of the polymorphisms *FAS* -670A/G and *FASL* -124A/G in individuals with tuberculosis (TB) and the control group (CG).

Genotypes and Alleles	TB *n* = 200 *n* (%)	CG *n* = 200 *n* (%)	*p* ^†^
***FAS* -670A/G**			
AA	30 (15.0)	36 (18.0)	0.4294
GA	88 (44.0)	94 (47.0)	
GG	82 (41.0)	70 (35.0)	
*A	0.37	0.41	0.2184
*G	0.63	0.59	
***FASL* -124A/G**			
AA	168 (84.0)	162 (81.0)	0.5106
AG + GG ^#^	32 (16.0)	38 (19.0)	
*A	0.91	0.9	0.5418
*G	0.09	0.1	

*n*: number of individuals; * Allele; ^#^ Absolute frequency of GG was insufficient for statistical analysis; ^†^ Chi-square test.

## Data Availability

The data analyzed in this study are included within the paper.
